# BRAF-activated lncRNA predicts gastrointestinal cancer patient prognosis: a meta-analysis

**DOI:** 10.18632/oncotarget.14061

**Published:** 2016-12-21

**Authors:** Yang-Hua Fan, Min-Hua Ye, Lei Wu, Miao-Jing Wu, Shi-Gang Lu, Xin-Gen Zhu

**Affiliations:** ^1^ Department of Neurosurgery, The Second Affiliated Hospital of Nanchang University, Nanchang 330006, Jiangxi Province, People's Republic of China

**Keywords:** BANCR, long non-coding RNA, gastrointestinal cancer, biomarker, meta-analysis

## Abstract

BRAF activated non-coding RNA (BANCR) is often dysregulated in cancer. We performed a meta-analysis to clarify its functions as a prognostic indicator in malignant tumors. We searched the PubMed, Medline, OVID, Cochrane Library, and Web of Science databases to identify BANCR-related studies. Nine original studies and 898 total patients were included in the meta-analysis. Hazard ratios (HR) and 95% confidence intervals (CI) were extracted from the included studies to determine the relationship between BANCR expression and patient overall survival (OS). Odds ratios (OR) were calculated using RevMan 5.3 software to assess associations between BANCR expression and pathological parameters. High BANCR expression correlated with lymph node metastasis (LNM) (OR = 3.41, 95% CI: 1.82–6.37, *P* = 0.0001), distant metastasis (DM) (OR = 2.98, 95% CI: 1.76–5.07, *P* < 0.0001), tumor stage (OR = 3.11, 95% CI: 1.89–5.12, Z = 3.25, *P* < 0.0001), and poor OS (pooled HR = 1.98, 95% CI: 1.20–3.27, *P* = 0.008) in gastrointestinal (GI) cancer patients, but not in non-GI cancer patients. Our results support the notion that BANCR as a promising prognostic biomarker in Chinese patients with GI cancer.

## INTRODUCTION

Long noncoding RNAs (lncRNAs) are transcribed RNA molecules > 200 nucleotides (nt) in length that lack an open reading frame [1]. lncRNAs have important functions in disease, including epigenetic, transcriptional, and posttranscriptional regulation [2]. Recent studies have reported lncRNA dysregulation in various cancer types [3–6]. Some lncRNAs play roles in cancer progression, promoting or inhibiting proliferation, invasion and metastasis [7–8], and many are promising prognostic markers [9]. Approximately 8.2 million people die from malignant tumors and 14.1 million people are diagnosed with cancer worldwide each year [10]. According to the American National Center for Health Statistics, approximately 600 thousand Americans will die of cancer in 2016 [11]. Novel biomarkers, such as lncRNAs, that enhance prognostic accuracy and aid in therapeutic decision-making are essential for improving cancer patient outcomes.

BRAF-activated lncRNA (BANCR), a 693-nt lncRNA encoded on human chromosome 9, was discovered by McCarthy [12] and Flockhart [13] and colleagues in 2012. BANCR is highly expressed in melanoma and promotes melanoma cell migration [12–13]. Evidence suggests that BANCR might play important roles in cancer growth and metastasis [14] and may be a prognostic indicator in certain cancers. However, most studies reported so far have been limited by discrete outcome and sample size. We performed an updated meta-analysis to determine the prognostic value of BANCR in cancer patients.

## RESULTS

### Literature search analysis

**Figure 1:**
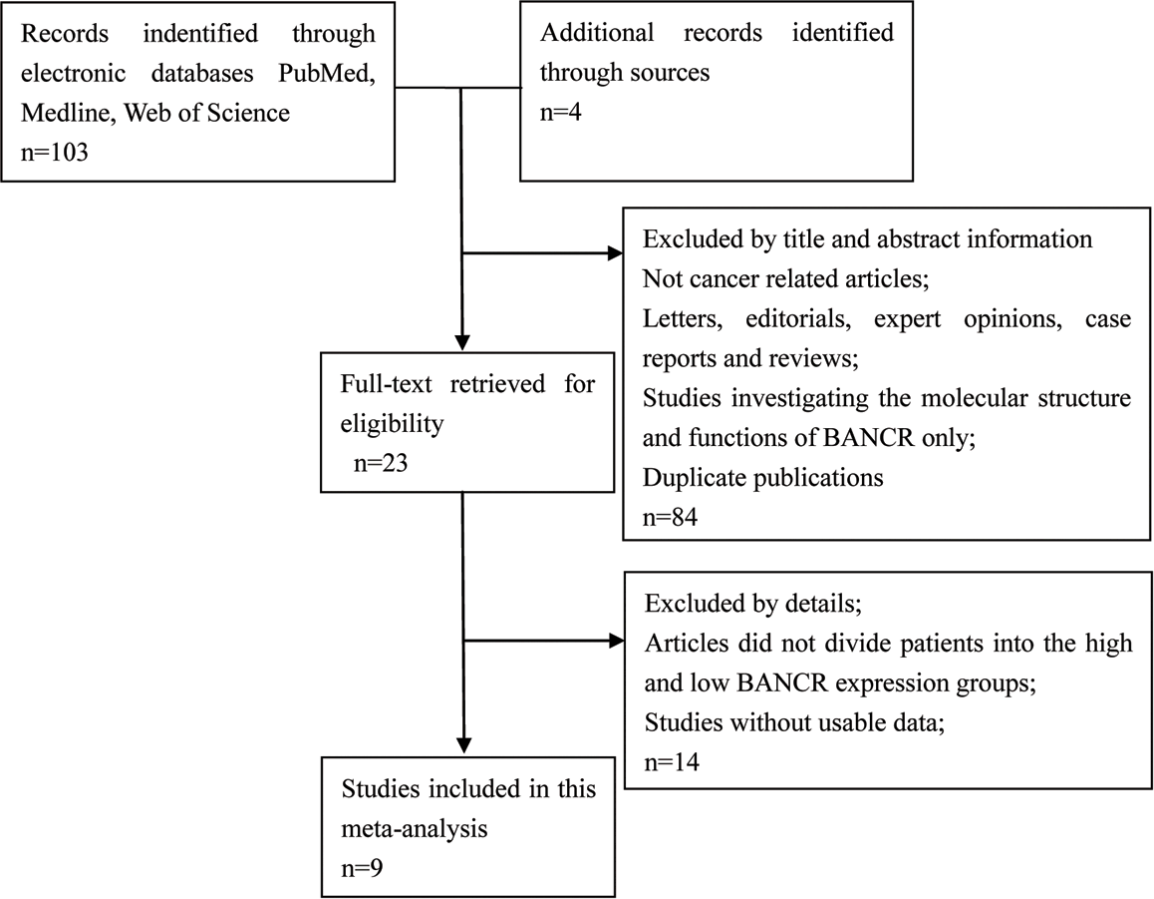
Flowchart depicting the literature search process, and study selection criteria

The detailed BANCR study screening process is shown in Figure 1. Based on inclusion and exclusion criteria, nine studies and 898 patients were included in the meta-analysis (Table 1) [15–23]. An average number of 99.8 subjects were analyzed in each study (range, 54 to 184). All studies were conducted in China and were published between 2014 and 2016. The included studies focused on colorectal cancer (CRC) [15], bladder cancer (BC) [16], gastric cancer (GC) [17], papillary thyroid cancer (PTC) [18], esophageal squamous cell carcinoma (ESCC) [19], osteosarcoma (OSC) [20], retinoblastoma (RB) [21], non-small cell lung cancer (NSCLC) [22], or hepatocellular carcinoma (HCC) [23]. BANCR expression was measured in tumor specimens. Lymph node metastasis (LNM), distant metastasis (DM), and tumor stage assessments depended on individual pathology. The Newcastle-Ottawa Scale (NOS) confirmed that all studies were of good quality (Table 2).

**Table 1:** Basic characteristics of all studies included in the meta-analysis

Study	Year	Region	Tumor type	Sample size	BANCR expression						Outcome	Survival Analysis	HR(95% CI)	Method
					High			Low						
					Total	LNM	DM	Total	LNM	DM				
Guo[15]	2014	China	CRC	60	18	14	−	42	16	−	−	−	−	qRT-PCR
He [16]	2016	China	BC	54	19	1	−	35	9	−	−	−	−	qRT-PCR
Li [17]	2015	China	GC	184	92	60	12	92	43	0	OS	Multivariate	1.511 (1.025−2.227)	qRT-PCR
Liao [18]	2016	China	PTC	92	29	14	−	63	30	−	−	−	−	qRT-PCR
Liu [19]	2016	China	ESCC	142	71	57	30	71	33	19	OS	Multivariate	2.238 (1.052−4.762)	qRT-PCR
Peng [20]	2016	China	OSC	84	42	−	20	42	−	10	OS	Multivariate	2.934 (1.123–7.664)	qRT-PCR
Su[21]	2015	China	RB	60	30	−	−	30	−	−	OS	Multivariate	2.903 (1.049–8.036)	qRT-PCR
Sun[22]	2014	China	NSCLC	113	53	19	−	60	40	−	OS	Multivariate	0.496 (0.262–0.938)	qRT-PCR
Zhou[23]	2016	China	HCC	109	54	−	−	55	−	−	OS	Multivariate	4.245 (1.324−13.609)	qRT-PCR

Note: The dashes represent no data.

Abbreviations: CRC, colorectal cancer; BC ,bladder cancer; GC, gastric cancer; PTC ,papillary thyroid cancer; ESCC, Esophageal Squamous Cell Carcinoma; OSC, osteosarcoma; RB ,retinoblastoma; NSCLC, non-small cell lung cancer; HCC, hepatocellular carcinoma; OS, overall survival; LNM, lymph node metastasis; DM, distant metastasis.

**Table 2:** Study quality was assessed according to the newcastle-ottawa scale

Author	Country	Adequate of case definition	Representativeness of the cases	Selection of Controls	Definition of Controls	Comparability of cases and controls	Ascertainment of exposure	Same method of ascertainment	Non-Response rate
Guo	China	٭	٭	٭	NA	٭٭	٭	٭	NA
He	China	٭	٭	٭	NA	٭٭	٭	٭	NA
Li	China	٭	٭	٭	٭	٭٭	٭	٭	NA
Liao	China	٭	٭	٭	NA	٭٭	٭	٭	NA
Liu	China	٭	٭	٭	٭	٭٭	٭	٭	NA
Peng	China	٭	٭	٭	٭	٭٭	٭	٭	NA
Su	China	٭	٭	٭	NA	٭٭	٭	٭	NA
Sun	China	٭	٭	٭	٭	٭٭	٭	٭	NA
Zhou	China	٭	٭	٭	NA	٭٭	٭	٭	NA

Notes: The quality of the article is through the number of stars(*).Through the selection and exposure categories, each numbered item gets a star at most. A maximum of two stars can be given for comparability. NA: not available.

### Association between BANCR expression and OS

**Figure 2:**
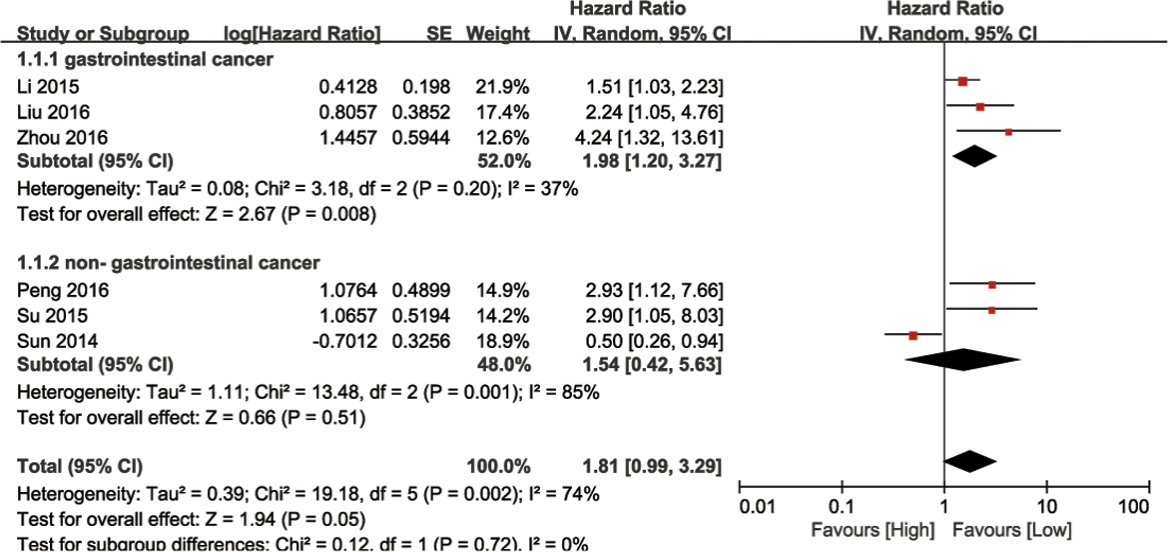
Forest plot showing the association between OS and BANCR expression in different cancer types

We performed a cumulative meta-analysis to assess the role of BANCR in cancer patient overall survival (OS). Six of the nine included studies with 645 total patients reported the relationship between OS and BANCR. The random effects model was used due to significant heterogeneity (I^2^ = 74%, *P* = 0.002). The HR for the high BANCR expression group versus the low expression group was 1.81 (95% CI: 0.99–3.29, *P* = 0.05; Figure 2). Due to the presence of heterogeneity, subgroups were analyzed based on cancer type (gastrointestinal [GI] or non-GI cancer). This revealed an association between BANCR and OS in GI cancer patients (HR = 1.98, 95% CI: 1.20–3.27, *P* = 0.008), but not between BANCR and non-GI cancer patients (HR = 1.54, 95% CI: 0.42–5.63, *P* = 0.51). There was no significant heterogeneity among GI cancer studies (I^2^ = 37%, *P* = 0.20). In a sensitivity analysis of all six studies, heterogeneity disappeared after the Sun, *et al*. study [22] was excluded (I^2^ = 16%, *P* = 0.31). BANCR and OS were associated (HR = 1.94, 95% CI: 1.44–5.62, *P* < 0.0001) in the five remaining studies. These results demonstrated that high BANCR expression might correlate with shorter OS in GI cancer patients in China, and BANCR was an independent factor for OS in these patients.

### Association between BANCR expression and LNM

**Figure 3:**
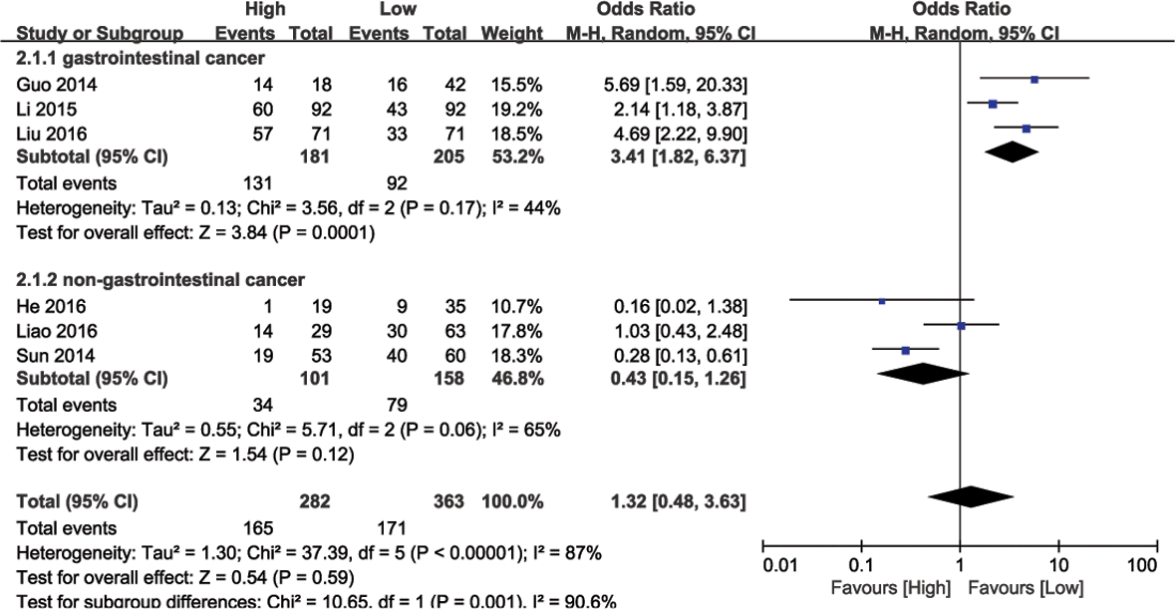
Forest plot showing the association between LNM and BANCR expression in different cancer types

Data from 645 cancer patients in six eligible studies were collected and analyzed. The random effects model was used due to significant heterogeneity (I^2^ = 87%, *P* < 0.00001). Meta-regression analysis and subgroup analysis (GI or non-GI cancer) were performed to explore heterogeneity sources. In the subgroup analysis, BANCR and LNM were associated in GI cancers (OR = 3.41, 95% CI: 1.82–6.37, *P* = 0.0001), but not in non-GI cancers (HR = 0.43, 95% CI: 0.15–1.26, *P* = 0.06; Figure 3). There was no significant heterogeneity among GI cancer studies (I^2^ = 44%, *P* < 0.17). These results demonstrated that high BANCR expression predicted a higher likelihood of LNM in GI cancer patients.

### Association between BANCR expression and DM

**Figure 4:**
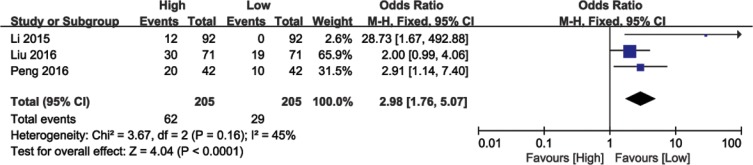
Forest plot showing the association between DM and BANCR expression in different cancer types

Analysis of 410 patients from three eligible studies demonstrated association between BANCR levels and cancer patients with DM (Figure 4). Analysis by the fixed effects model showed no significant heterogeneity (I^2^ = 45%, *P* = 0.16) and the pooled OR was 2.98 (95% CI: 1.76–5.07, *P* < 0.0001; high versus low BANCR; Figure 4). As a result, patients with DM clustered in the high BANCR expression group. These results indicated that high BANCR expression predicted increased probability of DM.

### Association between BANCR expression and tumor stage

**Figure 5:**
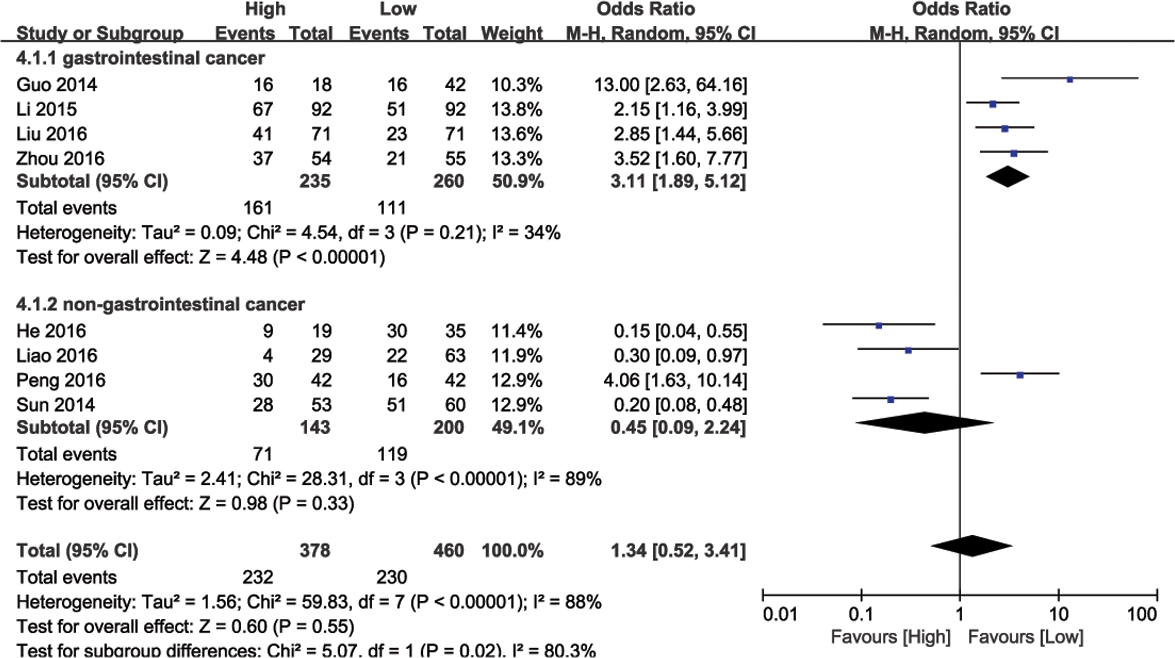
Forest plot showing meta-analysis of BANCR with respect to tumor stage in different cancer types

The relationship between BANCR expression and tumor stage was analyzed in 692 patients from eight eligible studies. Due to significant heterogeneity among the studies (I^2^ = 88%, *P* < 0.00001), the random effects model was used to calculate the pooled HR with corresponding 95% CI. The OR of the high BANCR expression group versus the low expression group was 1.34 (95% CI: 0.52–3.41, *P* = 0.55; Figure 5). Subsequent subgroup analyses were performed based on cancer type. Higher BANCR expression corresponded with higher tumor grade, with a pooled OR of 3.11 (95% CI: 1.89–5.12, *P* < 0.00001; Figure 5), with no obvious heterogeneity (I^2^ = 34%, *P* = 0.21) in GI cancer studies. However, significant heterogeneity existed across non-GI cancer studies (I^2^ = 89%, *P* < 0.00001), and BANCR was not associated with tumor stage in these cancers (HR = 0.45, 95% CI: 0.09–2.24, *P* = 0.06; Figure 5). Our results demonstrated that higher BANCR expression predicted higher tumor stage in GI cancer patients in China.

### Publication bias

**Figure 6:**
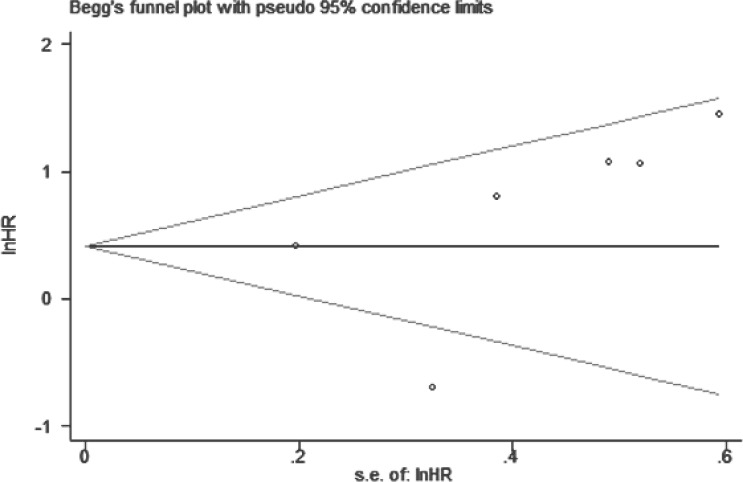
Funnel plot to assess publication bias with respect to the association of BANCR with OS in different cancer types

For our meta-analysis of the association between BANCR expression and OS, Begg's funnel plot and Egger's test results (*P* > |t| = 0.366, 95% CI: -3.643–7.867) showed no significant publication bias across the included studies (Figure 6).

## DISCUSSION

The occurrence of metastasis in cancers indicates poor patient prognosis and hence is an important survival indicator [24–25]. In addition, LNM and DM are important for cancer patient TNM (tumor–node–metastasis) staging, and for predicting patient prognosis. Since the precise metastasis mechanisms remain unknown in most cancers, molecular biomarkers play critical roles in cancer diagnosis, prognosis and treatment [26–27]. Finding new molecular markers that accurately predict tumor metastasis risk are of paramount importance for improving patient outcomes.

Mammalian genome analyses suggest that more than 80% of transcription is associated with lncRNAs [28]. lncRNAs play central roles in the regulation of cell differentiation, development, and proliferation [29]. Due to specific lncRNA associations with tumor development, and their presence in body fluids and tumor tissues, lncRNAs are promising biomarkers for tumor diagnosis and monitoring [30].

BRAF-activated lncRNA (BANCR) was discovered in melanoma, and promotes melanoma cell migration [12]. BANCR appears to be associated with tumorigenesis, and is dysregulated in colorectal, bladder, gastric, papillary thyroid, and lung cancer, and in ESCC, osteosarcoma, and hepatocellular carcinoma [15–23]. However, assessments of the prognostic value of BANCR in cancer patients were contradictory and inconclusive. On one hand, BANCR knockdown inhibited the malignant progression of melanoma, colorectal cancer and osteosarcoma [12, 15, 20, 31]. These studies suggest that BANCR acts as an oncogene to promote tumor growth. However, BANCR may function as a tumor suppressor in lung cancer [22, 32], and conclusions from two papillary thyroid cancer studies were contradictory [15, 33]. Because the role of BANCR as a molecular biomarker in human cancer was unclear, our study explored the prognostic value of BANCR in cancer patients via a meta-analysis.

Subgroup analyses in a fixed or random model allowed us to assess the role of BANCR in different cancer types. Our data showed that higher BANCR expression was indicative of advanced disease and poor prognosis in patients with GI cancer. By combining HRs from Cox multivariate analyses, we observed reduced GI cancer patient OS in high BANCR expression groups as compared to low expression groups (pooled HR = 1.98, 95% CI: 1.20–3.27, *P* = 0.008). Higher BANCR expression was also associated with LNM (OR = 3.41, 95%CI: 1.82–6.37, *P* = 0.0001), DM (OR = 2.98, 95% CI: 1.76–5.07, *P* < 0.0001), and tumor stage (OR = 3.11, 95% CI:1.89–5.12, *P* < 0.00001) in these patients. These associations were not observed in non-GI cancer patients.

Certain limitations must be taken into account when interpreting the conclusions of our meta-analysis. First, all studies were from China. Therefore our data may not represent global populations. Second, the included cancer types and patient numbers were small. Third, the criterion for high BANCR expression varied for different studies. Additional studies are needed to confirm the function of BANCR in various cancers.

In conclusion, high BANCR expression was associated with LNM, DM, advanced tumor stage, and poor OS in multiple GI cancers. Our results support BANCR as a promising prognostic biomarker for GI cancer patients in China.

## MATERIALS AND METHODS

### Literature search to identify relevant studies for meta-analysis

According to standard meta-analysis guidelines [34–35], two authors (Yang-Hua Fan and Min-Hua Ye) independently performed systematic searches for relevant articles concerning BANCR as a prognostic biomarker for cancer patient survival via the online databases, OVID, PubMed, Medline, and Web of Science. The latest search was updated on October 10, 2016. We searched using both text and MeSH terminology approaches, with the terms ‘BANCR’, ‘BRAF activated non-coding RNA’, ‘long intergenic noncoding RNA’, ‘lncRNA’, ‘noncoding RNA’, ‘cancer’, tumor’, carcinoma’, neoplasm’, ‘prognostic’, ‘prognosis’, ‘outcome’, ‘survival’ or ‘recurrence’. The strategy was adjusted for each database to maximize chances of finding the appropriate articles. Manual searches were also performed using the reference lists of relevant articles.

### Selection criteria for study inclusion

Two researchers, Yanghua Fan and Shi-Gang Lu, evaluated all included studies and extracted the data independently. Criteria used to include studies in the meta-analysis were as follows: 1) BANCR expression was measured in human tumor tissue, and patients were grouped according to BANCR level; 2) all tumors were confirmed by pathological or histological examinations; 3) studies statistically analyzed patient overall survival or pathological parameters such as LNM, DNM, and tumor stage, with respect to BANCR expression.

Exclusion criteria included: 1) articles that were reviews, letters, editorials, case reports and expert opinions; 2) non-English language and non-human studies; 3) studies lacking data listed in the inclusion criteria; 4) basic BANCR characterization studies.

### Data extraction from relevant studies

Two reviewers, Yanghua Fan and Lei Wu, independently extracted and examined data from the selected original articles. Any literature assessment disagreements were resolved through a third reviewer, Xingen Zhu. The following details were collected from each study: first author surname, publication year, country, tumor type, sample size, number of patients with lymph node and distant metastasis, BANCR HR and 95% CI for OS, and BANCR detection method.

### Statistical analysis

Study quality was assessed according to the Newcastle-Ottawa Scale (NOS). Statistical analyses were performed using RevMan version 5.3 software. Heterogeneity among different studies was measured by the *Q* and *I*^2^ tests. A probability value of I^2^ ≥ 50%, and *P* < 0.1 indicated significant heterogeneity [36]. A random effects or fixed effects model was used depending on heterogeneity analysis results. If there was significant heterogeneity among the studies, the random-effects model was adopted. Potential publication bias was assessed by the Begg's funnel plot through Stata 12.0 software. Pooled HRs and ORs were extracted from the published data. When HRs and 95% CIs were not directly reported, survival information was extracted from Kaplan-Meier curves and used to estimate the HR. The log HR and standard error (SE) were used to summarize overall survival outcome [37]. Odds ratios (ORs) and their 95% CIs were used to assess associations between BANCR expression and tumor parameters, including LNM, DM and tumor stage.
